# Extraction of a Foreign Body Implanted in the Uterus

**Published:** 1841-09

**Authors:** 

**Affiliations:** The Hospital St. Louis


*Extraction of a Foreign Body implanted in the Uterus.* By M. Maisonneuve, of the Hospital St. Louis.—This patient was thirty years of age on her admission to the hospital, Sept. 14, 1840. At the age of twenty-eight years she was in good health, became pregnant, and in the fifth month of gestation states that she miscarried and suffered from severe metro-peritonitis. She was obliged to enter the hospital La Pitié soon after she had begun to leave her room, where the surgeons diagnosticated metritis with hypertrophy of the anterior surface of the uterus. Various means were persevered in without effect, and when she came under the care of M. Maisonneuve her general powers were enfeebled, digestion bad, hectic at night, and she had dull continued pains in the loins and hypogastrium, which latter region was occupied by an irregular hard tumor, slightly painful on pressure, which filled the pelvis and extended into the iliac fossa. The os uteri permitted the entrance of the finger, but the body and neck of the organ were lost in an irregular, hard, and absolutely immovable mass. Examined by the speculum the vagina appeared to be in the normal state, and the only unusual appearance was abnormal patency of the os uteri, which permitted the surgeon to see something whitish. He passed a stylet to discover the nature hf this substance, and was astonished to find that he could circumscribe the unknown object by passing the stylet before and behind it. It adhered to the lips of the os uteri on all sides.

Persuaded that this was a foreign body implanted in the walls of the uterus, M. Maisonneuve first endeavored to divide it with scissors, but could not. He then placed one beak of a pair of long polypus forceps behind and the other before it, and by careful traction removed, without causing much pain, a piece of wooden stick, 122 millimeters in length, pointed at one extremity and bent at the other. Looking to her account of the case, it appeared highly probable that this stick had been broken in the uterus during criminal efforts to produce abortion, and this opinion has been since confirmed.

The operation was followed by a return of abdominal and lumber pains, and great febrile reaction, which disappeared in about eight days under general bleeding, baths, and several applications of leeches. But there remained a tumor in the pelvis which probably resulted from chronic adhesions of the uterus, bladder, and rectum, with some of the intestines. However, by the 1st of January, 1841, the tumor had greatly diminished and the general health was much improved.—*Ibid, from Gazette Medicale de Paris. Avril* 3, 1841.

